# Stochasticity in the symmetric division of plant cells: when the exceptions are the rule

**DOI:** 10.3389/fpls.2014.00538

**Published:** 2014-10-09

**Authors:** Sébastien Besson, Jacques Dumais

**Affiliations:** ^1^Centre for Gene Regulation and Expression, College of Life Sciences, University of DundeeDundee, UK; ^2^Facultad de Ingeniería y Ciencias, Centro de Innovación en Bioingeniería, Universidad Adolfo IbáñezViña del Mar, Chile

**Keywords:** Errera's rule, cell division, cytoskeleton dynamics, stochasticity, minimal area

Long before most of the molecular aspects of cell division were uncovered, L. V. Heilbrunn remarked that: “it is easier to make a new theory of cell division than to test an old one” and promised his readers to limit his treatment of cell division to “the factual facts regarding the physical changes which take place during mitosis” (Heilbrunn, 1928). Heilbrunn's barb was directed, first and foremost, to the theories or empirical rules put forward to explain how cells select a plane of division. A perusal of the cell biology literature in the decades preceding Heilbrunn's comment suffices to appreciate the author's cynicism toward cell division theories. By the end of the nineteenth century, at least five different rules had been formulated to predict how cells select a division plane. Of these, the most widely cited rules for plant cells were the Rectangular Section formulated by Sachs (1878) and the Principle of Minimal Area promoted by Berthold (1886) although often attributed to Errera (1888). The presence of exceptions to these rules led to many more “improved” rules and fueled a heated debate that spilled well into the twentieth century.

An intriguing feature of this story is that despite the explosion of cell biology research in the twentieth century, the nineteenth century obsession with cell division rules rapidly receded; and ultimately vanished before the tests Heilbrunn so eagerly desired were performed. Although many biologists have cited this early work in reviews (e.g., Smith, 2001; Kwiatkowska, 2004; Dumais, 2007), the classical division rules have laid essentially dormant for a full century. The reasons why these rules were never tested must be sought in the particular mind-set of twentieth century biology. The most important factor is probably the geometrical nature of the rules which did not resonate well with the molecularly-oriented biology of the last century. Certainly, reducing cell division to a geometrical problem adds little to the “factual facts regarding the physical changes which take place during mitosis.” Yet, it is probable that even the most abstract division rule would not have been neglected for so long if it had predicted with great accuracy the selection of division planes in plant cells. Thus, another factor seems to have played an important role: the fact that even within the confine of a specific tissue, cell division seems to escape the determinism embodied by the classical rules. The frequent exceptions to the predicted division planes must have invalidated the division rules to the eye of most biologists. We recently argued that these exceptions may in fact be confirmation of another, more subtle, division rule (Besson and Dumais, 2011). Here we briefly retrace the history leading to this new rule while, at the same time, highlighting the strange turn of events that greatly delayed the acceptance of stochasticity in this particular area of cell biology.

## Cell division and soap films

**Figure 1:**
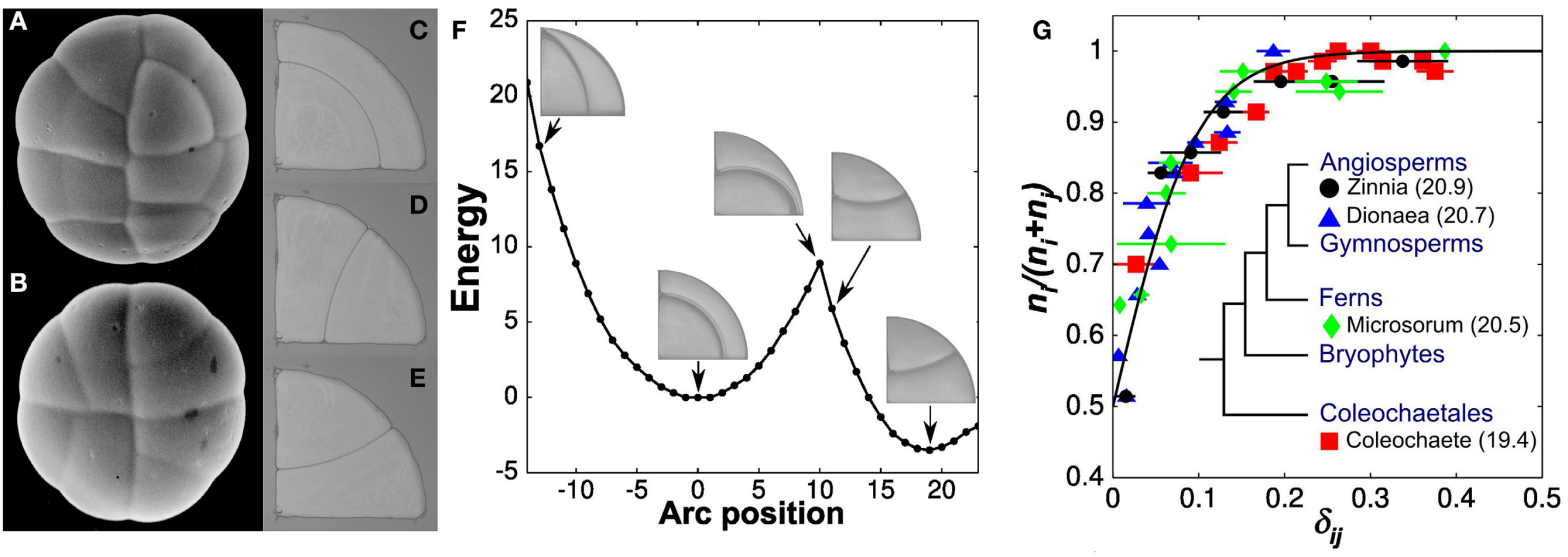
**Stochastic rule for the selection of a division plane. (A,B)** Two glandular trichomes showing the quadrant cells studied by Berthold and Thompson. Each circular gland divides twice across its center to produce four quadrant cells. These quadrant cells can undergo three types of division, all of which are predicted by soap films **(C–E)**. The division plane with least area is anticlinal, forming a wedge cell and a triangular cell **(D,E)**. The other division is periclinal **(C)** and corresponds to a local minimum of surface area or surface energy in the case of soap bubbles **(F)**. **(G)** The proportion of cells adopting the optimal division plane *i* as a function of the relative area difference, δ_*ij*_, in its favor. The solid line is the best fit of the experimental data with a probability function of the form (1 + exp(-βδ_*ij*_))^−1^. Values of β computed for individual species are also reported (inset) (modified from Besson and Dumais, 2011).

The most perennial theory for cell division was inspired by the properties of soap films as described by Plateau (1873). In a singular paper, published both in French and German, Errera states: “a cell wall, at the moment of its formation, tends to assume the form which a weightless liquid film would assume under the same conditions” (Errera, 1886, 1888). Errera draws several conclusions from his new principle, most importantly that division planes ought to be surfaces of constant mean curvature and that Sachs' rectangular section is a natural consequence of his rule. However, one conclusion that Errera appears to avoid purposely is the idea that the dividing wall, being subjected to the same surface tension as soap films, will invariably assume the configuration of minimal surface area. Errera's omission is in sharp contrast with statements made at the same time by Berthold (1886) who insisted on the minimization of area. One finds an explanation for the deliberately broad definition adopted by Errera in the work of one of his student, de Wildeman (1893). de Wildeman follows Errera's soap film analogy but now recognizes the possibility of multiple division planes for a given cellular morphology. He says: “When new walls, under the influence of their own tension, are fashioned into surface of constant or zero mean curvature; they represent minimal surfaces. But it is a relative minimum rather than an absolute minimum, as clearly indicated by Plateau. *It is therefore not necessary that all new walls in a cell occupy, of all the possible positions, the one of least area*” (p. 9). Later in his monograph, de Wildeman returns to this theme—“*the dividing wall possesses a fairly large leeway in adopting its form*. In fact, it will find itself in a stable equilibrium as long as its surface is a *relative* area minimum, its mean curvature is constant and it attaches itself to the surrounding wall at right angle…” (p. 76). The quadrant cell, a favorite of Berthold (1886), illustrates well the different relative area minima that are possible within a cell and thus the “fairly large leeway” a cell possesses in selecting a division plane (Figures 1A–F). It is striking that Errera and de Wildeman recognized the co-existence of multiple division planes for a given cell geometry and therefore excluded in very explicit terms a deterministic interpretation of their division rule. This subtle view of plant cell division, however, was not meant to persist very long.

## The principle of minimal area

Ironically, the nuanced version of Errera's rule seems to have been lost because of a desire to check its predictive power. To be tested, Errera's rule had to move beyond the level of a soap film analogy to a fully operational definition giving explicit predictions for specified cellular geometries. Thus, Berthold's principle of minimal area came to be the incarnation of Errera's rule whenever predictions had to be made. This tension is clearly seen in D'Arcy Thompson's book “On Growth and Form” (Thompson, 1917). Thompson first states Errera's rule following precisely the soap film analogy used by Errera himself (p. 306) but as he prepares to calculate the division plane of an idealized quadrant cell, he promptly equates Errera's rule to Berthold's minimal area principle: “the incipient partition-wall of a dividing cell tends to be such that its area is the least possible by which the given space-content can be enclosed.” (p. 348). Following Thompson's work, the great majority of references to Errera's rule are cast in terms of an absolute area minimum; while the 1886 formulation and its nuanced interpretation were revisited only on a handful of occasions in the twentieth century. Wardlaw must be credited for the final and clearest statement of the spirit of Errera's rule, which appeared in a book commemorating the centennial of Leo Errera's birth:

“In essence, Errera was calling attention to fundamental physiological and physical relationship, the relationship so aptly described in the title of his classical paper—a fundamental condition of equilibrium in living cells. In this general approach we are reminded of the writings of Plateau, from which Errera's idea was derived; for Plateau stated quite clearly that a surface of minimal area was to be understood as a *relative* minimum, determined as it would be by all the material agencies. If, therefore, we accept Errera's Law in its general aspect (as he himself clearly intended), recognizing that in different biological circumstances other factors will inevitably become incident and may affect the pattern of wall formation to a greater or less extent, then the value of the Law, far from being diminished by seeming exceptions, is enhanced by them” (Wardlaw, 1960).

It is probably fair to say that Wardlaw's lucid reflections, just as those of Errera and de Wildeman, left no mark on the field of cell biology until stochasticity forced itself into experiments.

## Revisiting stochasticity

The first inkling of the importance of stochasticity in cell division came from studies of the morphogenetic transitions in fern protonemata; most prominently the analysis by Cooke and Paolillo of division plane selection in the apical cell of protonemata (Cooke and Paolillo, 1980, see also Miller, 1980). Cooke and Paolillo's data show that the transverse and longitudinal division planes co-exist for a broad range of cell geometries but are expressed in different proportions depending on the aspect ratio of the apical cell. Thirty years later, Dupuy et al. (2010) obtained similar results for the marginal cells of *Coleochaete*.

By demonstrating broad transitions between different modes of cell division, these works paved the way for a fully stochastic division rule in which two or more division planes, each representing a *local* area minimum, compete to express themselves in a given cell geometry. However, an important question remained: can a unique stochastic model explain the selection of a division plane in a broad range of plant systems? We set out to answer this question in 2007, after having rediscovered de Wilderman's alternative division planes while preparing a figure for a review paper (Dumais, 2007). Our results showed that the competing division planes are observed according to a precise probability function and, more importantly, that this function is conserved across a wide sampling of plant systems (Besson and Dumais, 2011) (Figure 1G). Therefore, although it is impossible to predict the *exact* way a cell will divide, it is possible the compute precisely the possible division planes and their respective probability. Using this new information, we reformulated Errera's rule as: *symmetrically dividing plant cells select a division plane from a set of minimal area configurations according to an exponential probability distribution that increases inversely with the surface area of the configurations*. This definition formalized the rule described by Errera and de Wildeman more than 100 years earlier.

## The origin and role of stochasticity

This account of plant cell division has left out one important side of the story—the sustained effort to uncover the molecular underpinnings of mitosis. Two questions arise naturally if one accepts Errera's rule for plant cell division. First, what components or properties of the cell division machinery allow the cell plate to behave like a soap film? Second, what is the molecular basis of the stochasticity observed at the cellular level? The first question was addressed by Lloyd, Venverloo and co-workers who provided evidence that tense cytoskeletal elements projecting from the nucleus and interacting with the pre-prophase band can explain many of the features associated with the positioning of the division plane (Venverloo and Libbenga, 1987; Lloyd, 1991). Based on this seminal work, we have argued that the stochasticity observed at the cell level is a reflection of the stochasticity inherent to the cytoskeletal dynamics supporting the selection of the division plane. In particular, we showed that the observed division planes correspond to the most probable distribution of tense microtubular strands competing over a fixed pool of tubulin (Besson and Dumais, 2011). Thus, the stochasticity observed at the cell level is only a reflection of the dynamic instability of microtubules that allows them to “sense” a cell's geometry by exploring several configurations spanning the entire cell volume. Such conclusion forces us to shift perspective on the role of stochasticity in cell biology. Stochasticity does not always reflect a lack of regulation; it can also *be* the mechanism of regulation. Without dynamic remodeling of the cytoskeleton, cells could not sense their shape and therefore would be unable to align their division plane. Other examples of stochasticity playing a constructive role in biology have been demonstrated in the perception of weak signals by biological sensors (Wiesenfeld and Moss, 1995) and the “search and capture mechanism” allowing the spindle to attach to chromosomes (Holy and Leibler, 1994).

## Overcoming stochasticity

If the underlying mechanism for the selection of a division plane is intrinsically stochastic, how can we explain the regularity of developmental events? To answer this question, it is important to realize first that stochasticity does not mean absence of control. In that respect, a notable consequence of the stochastic division rule expounded above can be deduced directly from the exponential law that governs it (Figure 1G). While isodiametric cells (small δ_*ij*_ values) exert poor control over the selection of their division plane, highly elongated cells (large δ_*ij*_ values) adopt reliably the division plane of absolute minimal area and, as a result, invariably cut their long axis. The stochastic division rule thus approximates a deterministic rule whenever a cell deviates strongly from an isodiametric shape. This fact alone can explain a broad range of stereotypical cellular patterns even if the underlying mechanism is stochastic (Besson and Dumais, 2011).

As noted soon after the publication of Errera's paper, there are however several plant tissues where cell division deviates systematically from any division rule that one could formulate based on the behavior of soap films. Explicit examples are found in the division of cambial initials, the formation of stomatal complexes (Rasmussen et al., 2011) and early embryogenesis (Yoshida et al., 2014). All of these examples fit under the rubric of asymmetric cell division. Observations of asymmetric cell division suggest a level of control above a cell's own geometry. What those controls are remains unclear although auxin signaling (Yoshida et al., 2014) and mechanical stress (Uyttewaal et al., 2012; Louveaux and Hamant, 2013) are promising candidates. Thus, the emerging picture for the control of plant cell division is one where a stochastic division mechanism governs a broad range of symmetric cell divisions based solely on cell geometry; while a deterministic division mechanism, presumably based on global cues rather than local cues, takes over when the fulfillment of a specific cellular pattern is a necessity.

## Conclusion

This brief historical survey of the origin and evolution of division rules for plant cells highlights the difficulties in acknowledging stochasticity in biology. Fluctuations are an integral part of biology but are too often interpreted as masking underlying deterministic processes. As a result, the subtle division rule outlined by Errera was neglected for over one century because it implied stochasticity in the selection of a division plane. It is now clear that stochasticity can play a constructive role in the cell; either by supporting cytoskeletal dynamics (Holy and Leibler, 1994; Besson and Dumais, 2011) or allowing receptors to perceive faint signals (Wiesenfeld and Moss, 1995). We can expect many more examples to arise as cell biologists become more attuned to thinking in terms of stochasticity.

### Conflict of interest statement

The authors declare that the research was conducted in the absence of any commercial or financial relationships that could be construed as a potential conflict of interest.
